# cIAP1/2 are involved in the radiosensitizing effect of birinapant on NSCLC cell line in vitro

**DOI:** 10.1111/jcmm.16526

**Published:** 2021-05-03

**Authors:** Hao Sun, Yanan Du, Ming Yao, Qin Wang, Kaihua Ji, Liqing Du, Chang Xu, Ningning He, Jinhan Wang, Manman Zhang, Yang Liu, Yan Wang, Kaixue Wen, Qiang Liu

**Affiliations:** ^1^ Institute of Radiation Medicine Chinese Academy of Medical Sciences & Peking Union Medical College Tianjin China; ^2^ Tianjin Center for Disease Control and Prevention Tianjin China; ^3^ Shanxi Academy of Medical Sciences Shanxi Bethune Hospital Shanxi China

**Keywords:** apoptosis, birinapant, IAP family, NSCLC, radiosensitive

## Abstract

Tumour radioresistance is a major problem for cancer radiation therapy. To identify the underlying mechanisms of this resistance, we used human non‐small cell lung cancer (NSCLC) cell lines and focused on the Inhibitor of Apoptosis Protein (IAP) family, which contributes to tumourigenesis and chemoresistance. We investigated the possible correlation between radioresistance in six NSCLC cell lines and IAP protein levels and tested the radiosensitizing effect of birinapant in vitro, a molecule that mimics the second mitochondria‐derived activator of caspase. We found that birinapant‐induced apoptosis and inhibited the proliferation of NSCLC cells after exposure to radiation. These effects were induced by birinapant downregulation of cIAP protein levels and changes of cIAP gene expression. Overall, birinapant can inhibit tumour growth of NSCLC cell lines to ironizing radiation and act as a promising strategy to overcome radioresistance in NSCLC.

## 1 INTRODUCTION

Lung carcinoma is one of the most dangerous malignant tumours,^1^ and current treatments include surgery combined with radiotherapy and chemotherapy.^2^ The aim of the latter is to provide therapeutic doses to the target tumour volume while minimizing damage to normal adjacent tissues. However, it is difficult when tumours show high resistance to both radiation and drugs, thus weakening the therapeutic effect.^3^ More than 100 years ago, nitroimidazoles and anthraquinones were reported to sensitize some tumour cells to ionizing radiation (IR),^4^, ^5^ but the molecular mechanisms have yet to be identified.^6^, ^7^, ^8^

The Inhibitor of Apoptosis Protein (IAP) family are candidates for drug and IR resistance.^9^ For example, in lung cancer, X‐linked IAP (XIAP) is upregulated and contributes to inhibiting apoptosis.^10^ Indeed, XIAP is known to directly inhibit caspase proteins and is involved in the regulation of tumour proliferation and metastasis. However, mammals have eight IAPs,^11^ and the contribution of the other IAPs, for example, cellular IAP (cIAP), in tumour growth and resistance is unknown.^12^, ^13^, ^14^, ^15^, ^16^ cIAP1/2 has been shown to be overexpressed in lung cancer patients, and a higher expression is correlated with a worse prognosis, suggesting that cIAP1/2 is related to the occurrence and development of lung cancer and drug resistance.^17^

The baculoviral IAP repeat (BIR) domain, common to all IAPs, is required for caspase inhibition and protein complex formation. The RING domain (Really Interesting New Gene), located at the C‐terminus, functions as an E3 ligase. This domain also promotes dimerization and oligomerization, thus regulating ubiquitination.^18^ In the TNF‐α signalling pathway, cIAP1/2 oligomerizes and regulates its substrates such as RIP1 and NEMO, and activates NF‐κB signalling.^19^ Endogenous IAP antagonists, identified as Smacs (secondary mitochondrial‐derived caspase activators), use a peptide sequence (Ala‐Val‐Pro‐Ile) to inhibit IAP function by binding to their BIR domain, thus preventing IAP‐mediated inhibition of caspase proteins.^20^, ^21^, ^22^, ^23^ In order to promote tumour cell apoptosis, several IAP inhibitors have been designed that mimic the binding site of second mitochondria‐derived activator of caspase (Smac) and are currently undergoing investigation in early‐phase clinical trials. Several Smac mimetics have been reported to be effective in the treatment of a range of malignancies, such as LCL161 (Novartis) and birinapant in the treatment of triple‐negative breast cancer (TNBC).^24^, ^25^ In addition, Smac mimetics combined with radiation therapy seem to be a promising tumour treatment. For example, the bivalent Smac mimetic, BV6, binds to the BIR domain of IAP and induces IAP downregulation, activates apoptosis, and enhances radiosensitization of non‐small cell lung carcinoma in vitro.^26^, ^27^, ^28^ Human prostate cancer has been shown to be sensitized to radiation by the molecule SH‐130, one of the Smac‐mimetic IAP inhibitors.^29^ Birinapant can also increase the radiosensitivity of human head and neck cancer cells (HNSCC), but there is a lack of reports on its effects on other cell types.^30^

In the present study, we examined the correlation between radiosensitivity in non‐small cell lung cancer (NSCLC) cell lines and expression levels of cIAP1/2. In addition, we proved the possibility of birinapant as an effective radiosensitizer for lung cancer and analysed the contribution and possible mechanism of action of birinapant on NSCLC cell line radiosensitization. In order to clarify our conclusions, we used BV6, a common IAP antagonist as a positive control in a subset of experiments.

## 2 MATERIALS AND METHODS

### 2.1 Cell culture

Human NSCLC cell lines (H1299, H1650, H358, H460, A549 and HCC827) were obtained from the Tianjin Key Laboratory of Radiation Medicine and Molecular Nuclear Medicine & Institute of Radiation Medicine. Cells were cultured in RPMI1640 (Hyclone) supplemented with 10% bovine serum (Omega Scientific) at 37℃, 5% CO_2_ and 95% humidity.

### 2.2 Birinapant and BV6 treatment and irradiation procedure

The bivalent Smac mimetic birinapant was purchased from Medchemexpress (MCE, Medchemexpress CO. Ltd) and BV6 was purchased from Selleck (Blue Wood Chemical Co., Ltd.). Cells were treated with 10 µmol/L birinapant or BV6 as indicated, or with the equivalent amount of solvent dimethyl sulphoxide (DMSO, Beijing Dingguo Changsheng biotechnology CO. Ltd) 1 hour before irradiation. Irradiation with single doses of 2‐8 Gy was performed by a 137Cs γ‐source (Atomic Energy of Canada Limited, Gamma cell 40). The source‐target distance was 30 cm, and the dose rate was 0.99 Gy/min.

### 2.3 MTT assay

Cells were seeded at a density of 4 × 10^3^ cells per 200 µL in a 96‐well microplate, and cultivated for 24 and 48 hours after treatment with 10 µmol/L birinapant. Then, 10‐µL MTT (3‐(4,5‐dimethyl‐2‐thiazolyl)‐2,5‐diphenyl‐2‐H‐tetrazolium bromide) was added per well (5mg/mL). After 4 hours of incubation at 37℃ and 5% CO_2_, solubilization of the converted purple formazan was accomplished by the addition of 150 µL DMSO and vortexing for 10 minutes. The OD in each well was measured with a microplate reader (BioTek) at 570 nm.

### 2.4 Colony Formation Assay

Cells were seeded at a density of 1 × 10^3^ per 2 mL in a 6‐well microplate for 24 hours. After adhesion to the walls, they were incubated with 10 μmol/L birinapant or BV6 for 1 hour at 37℃ and 5% CO_2_. The plate was subsequently irradiated at room temperature by a 137Cs γ‐source, in the presence or in the absence of birinapant, and then were incubated in normal conditions for 7 days. Cells were fixed by methanol for 5 minutes and stained with 10% Giemsa (Sigma‐Aldrich) solution for 30 minutes. The plate was then gently washed in water, and the number of colonies containing 50 or more cells were counted. The colony formation rate (%) is the number of colonies divided by the number of incubated cells. A cell survival fraction curve was fitted based on the single‐hit‐multi‐target equation, and the curve parameter D_0_ was measured. The radiosensitization ratio (SER) was calculated as the ratio of D_0_ between the group exposed to the drug and irradiation, and the group just exposed to irradiation.

### 2.5 Cell death analysis

Apoptotic cells were detected by staining with Annexin V/propidium iodide (BD Biosciences‐US). The loss of plasma membrane is one of the earliest characteristics of apoptosis. At this time, the membrane phospholipid phosphatidylserine (PS) is transferred from the inner lobule of the plasma membrane to the outer lobule, thus exposing to the external environment of the cell. Annexin V is a Ca^2+^‐dependent phospholipid‐binding protein, which has a high affinity with PS and binds to cells exposed to PS. It can be used as a sensitive probe for the analysis of cells in the early stage of apoptosis by flow cytometry. FITC Annexin V staining precedes the loss of membrane integrity, which accompanies the latest stages of cell death resulting from either apoptotic or necrotic processes. Viable cells with intact membranes exclude PI, whereas the membranes of dead and damaged cells are permeable to PI. Cells were divided into four groups: control (no drug or irradiation), single‐drug group (only drug treatment), single‐radiation group (only radiation treatment), and combination group (both drug and radiation treatments). Each group of cells were seeded at a density of 2 × 10^5^ per 1 mL in a 6‐well microplate. After treatment (24 or 48 hours), cell death was detected by flow cytometry after the addition of 5µL of Annexin V and propidium iodide.

### 2.6 cIAP1 and cIAP2 protein and mRNA expression levels

Cells were collected at each time point, and cell lysates were prepared as described. Western blots were performed with the following antibodies: antibody to caspase‐3 (1:2500, ab32351, Abcam, US), cIAP1 (1:1000, ab154525, Abcam, US), cIAP2 (1:1000, ab32059, Abcam, US), β‐actin (1:5000, CW0096M, CWBIO, China), Goat Anti‐Mouse IgG, HRP Conjugated (1:5000, CW0102S, CWBIO, China), Goat Anti‐Rabbit IgG, HRP Conjugated (1:5000, CW0103S, CWBIO, China).

Cell RNA was extracted by Trizol^®^ (ambion, life, America) as described. mRNA expression levels were detected using the EvaGreen kit (abm, America) with 40 cycles. The program was set according to the manufacturer's standard protocol. Results were analysed with the 2^−ΔΔCt^ method. The GAPDH was used as the house‐keeping gene in all PCR experiments. Primers: cIAP1 (Forward) 5′‐TTGTCAACTTCAGATACCACTGGAG‐3′; (Reverse) 5′‐CAAGGCAGATTTAACCACAGGTG‐3′; cIAP2 (Forward) 5′‐TCCTGGATAGTCTACTAACTGCC‐3′; (Reverse)5′‐GCTTCTTGCAGAGAGTTTCTGAA‐3′.

### 2.7 Statistics data analysis

Statistical significance was tested with two‐sided unpaired Student's *t* tests and two‐way ANOVA, performed using Graphpad version 5.0 (GraphPad Software). Results were considered statistically significant for *P*‐values lower than 0.05.

## 3 RESULTS

### 3.1 Radiosensitivity and cIAP1/2 protein levels

**FIGURE 1:**
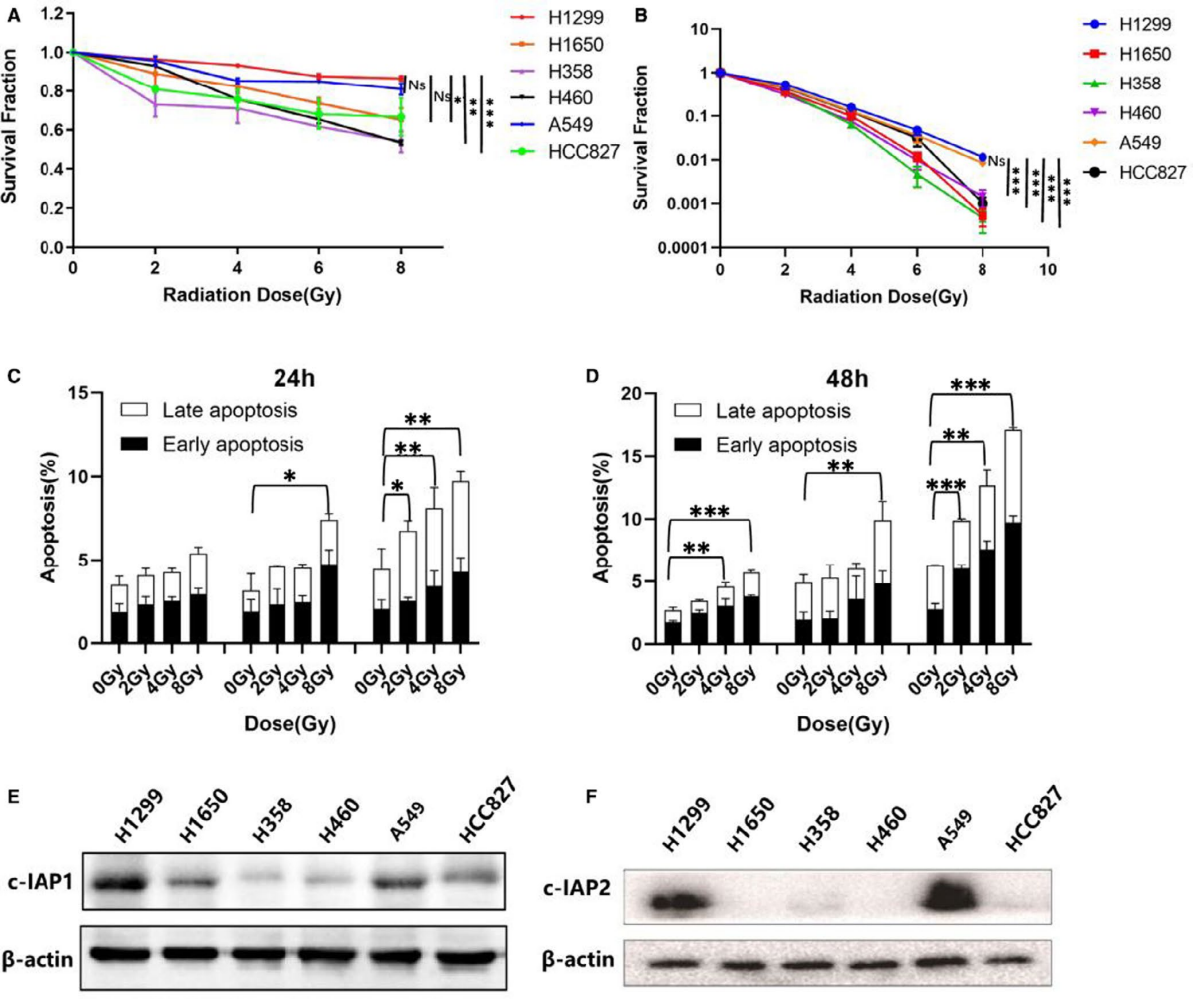
A, Cell viability was detected by MTT colorimetric method of IR on six NSCLC cell lines, 48 h after irradiation with γ‐rays at doses of 2, 4, 6 and 8 Gy; B, The clone formation ability of six non‐small cell lung cancer cell lines after ionizing radiation was observed by clone formation experiment; C, Apoptosis of lung cancer cell lines H1299, H460 and H1650, detected by flow cytometry, 24 h after IR exposure, respectively. H1650 cells, H1299 cells and H460 cells were represented from left to right; D, Apoptosis of lung cancer cell lines H1299, H460 and H1650, detected by flow cytometry, 48 h after IR exposure, respectively, H1650 cells, H1299 cells and H460 cells were represented from left to right; E, Western blot of cIAP1 protein expression levels in various cell lines. F, Western blot of cIAP2 protein expression levels in various cell lines. Error bars are means ± SD, n = 3 independent replicates and *P* < .05, *P* < .01, or *P* < .001 were considered statistically significant

An MTT colorimetric assay was used to study the impact of IR on cell proliferation After 48 hours of IR treatment, viability was reduced in all the cell lines tested (H1299, H1650, H358, H460, A549, and HCC827) in a dose‐dependent manner (Figure 1A). The effect was more pronounced in H460 and H358, whereas H1299 and A549 cells showed higher radiation resistance than other cell lines.

The radiosensitivity of six NSCLC cell lines was investigated using a colony‐forming assay (Figure 1B). After 7 days, the cloning ability decreased significantly, especially in the case of H460 and H358, whereas H1299 and A549 exhibited less sensitivity. Results for HCC827 were similar to those obtained in H1299 and A549 (except at the 8 Gy dose). Overall, H1299 and A549 cell lines were found to be the most resistant to IR treatment among these cell lines.

Apoptosis is one of the main ways of cell death caused by radiation.^31^ Three of the representative cell lines (H460, H1299, and H1650) were chosen to study the effect of irradiation on apoptosis (The preliminary results showed that H1299, H1650 and H460 had different radiation resistance (H1299 > H1650 > H460)). The effects were monitored 24 and 48 hours after irradiation (Figure 1C‐D, H1650 cells, H1299 cells and H460 cells were represented from left to right in each figure.). H460 cells were more sensitive to radiation than H1299 and H1650, consistent with the preliminary experiments. Subsequently, cIAP1 and cIAP2 protein levels were analysed by Western blot (Figure 1E‐F). The results showed that the expression of cIAP1 (some cells did not express the cIAP2 protein) seemed to be positively correlated with radiation resistance, that is, the more resistant to radiation the cells are, the higher expression level of cIAP1 proteins they have.

### 3.2 Changes of cIAP1/2 protein induced by radiation

**FIGURE 2:**
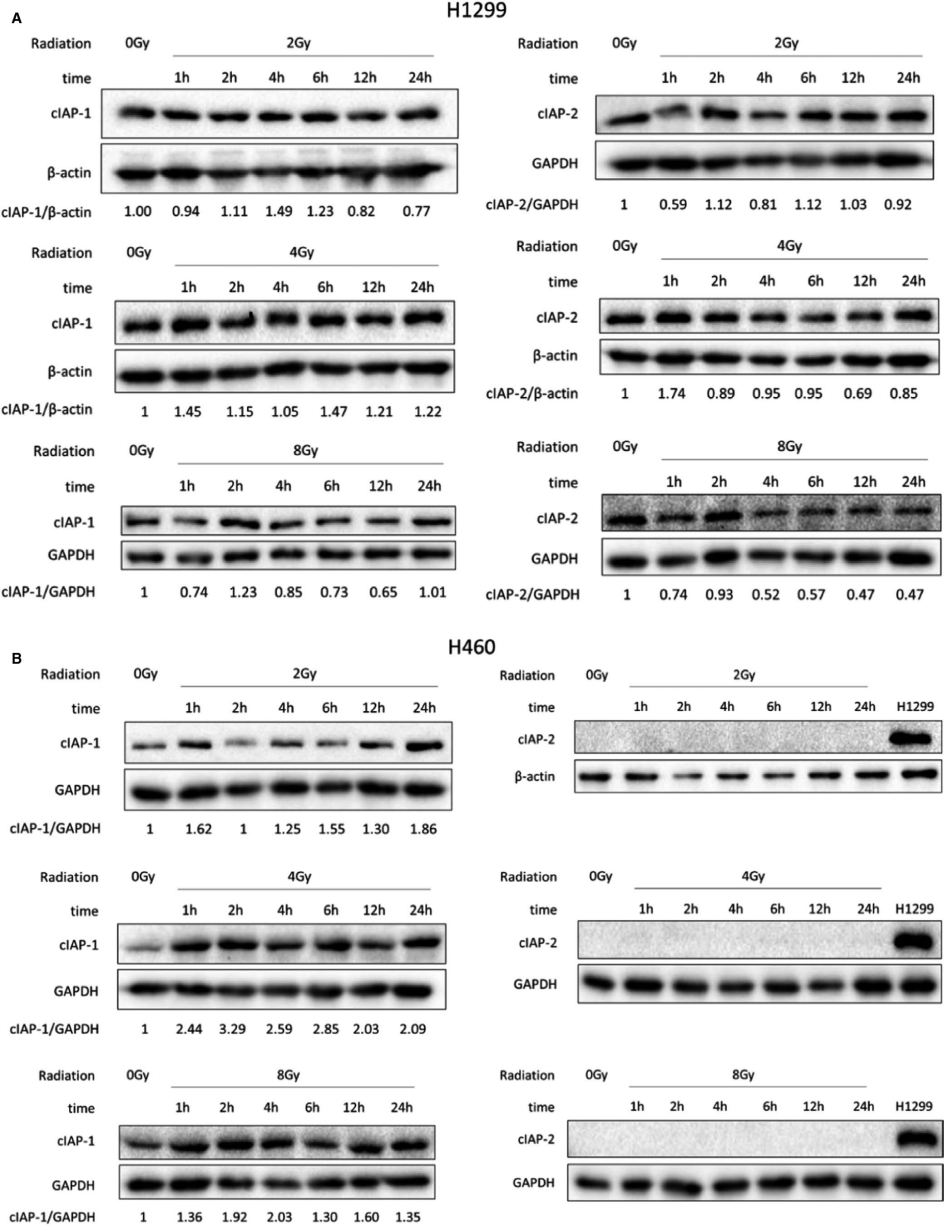
Western blot assays to determine the dose‐dependent effect of radiation on the expression levels of cIAP1/2 proteins in (A) H1299 cells and (B) H460 cells. All data shown are representative and have been repeated at least three times independently

Expression levels of cIAP1 were higher in IR‐resistant H1299 cells. Differences in cIAP2 protein levels were more evident than those observed for cIAP1. Expression of cIAP1 and cIAP2 was higher in cells resistant to IR, for example, H1299, than in those with low radioresistance, for example, H460. We chose these two cell lines, H1299 and H460, to study the effect of different doses of radiation on the expression of cIAP1 and cIAP2 at different time points (Figure 2A‐B). Figure 2 shows that although radiation‐induced changes in cIAP1/2 protein levels in H1299 cells were not significant, levels of cIAP1 protein in H460 cells were significantly increased, suggesting that cIAP1/2 expression is upregulated after radiation treatment. These results further confirm that cIAP proteins may play a positive role in cell radiation resistance.

### 3.3 Birinapant combined with radiation increased apoptosis of NSCLC cell lines

**FIGURE 3:**
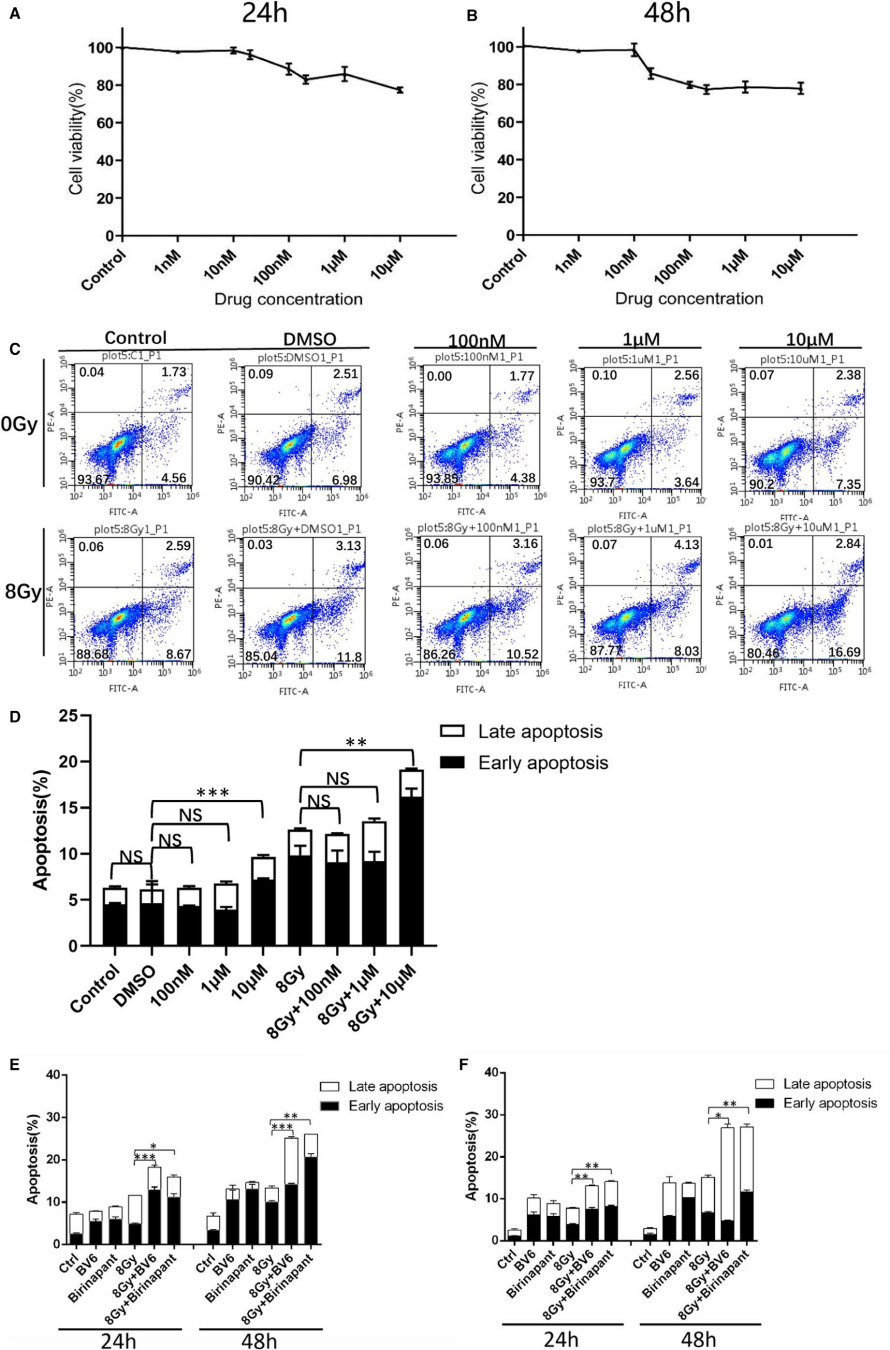
A, MTT colorimetric assay to test the toxicity of increasing concentrations of birinapant on H1299 cells after 24 h; B, MTT colorimetric assay to test the toxicity of increasing concentrations of birinapant on H1299 cells after 48 h; C, The effective concentration to induce apoptosis in H1299 cells was determined by flow cytometry after testing the indicated concentrations of birinapant after 24 h exposure; D, The statistical data of apoptosis of H1299 cells induced by different concentrations of birinapant by flow cytometry. E, apoptosis after different treatments of H1299 detected by flow cytometry; F, apoptosis after different treatments of H460 detected by flow cytometry. Data are presented as the means ± SD, n = 3 independent replicates, and *P* < .05, *P* < .01, or *P* < .001 were considered statistically significant

Since IAP proteins play an important role in the radioresistance of NSCLC cell lines,^28^, ^29^, ^30^ the IAP inhibitor birinapant was used. After H1299 cells were treated with different concentrations of birinapant in the total culture medium for 24 and 48 hours, the cell survival rate decreased steadily to 80% at 10 µmol/L (Figure 3A‐B).

First, we identified the effective birinapant concentration to induce apoptosis in H1299 after 24 hours treatment (Figure 3C‐D). Of the three concentrations tested, 100 nmol/L, 1 and 10 µmol/L, only 10 µmol/L birinapant treatment enhanced apoptosis. Thus, we used this concentration in subsequent experiments. The radiosensitization of birinapant was detected in H1299 and H460 cell lines. BV6, a similar kind of IAP inhibitor that has been proven to have a radiosensitizing effect, served as a positive control.^28^ A significant increase in apoptosis was observed in the combination group (drug and radiation exposure) compared to the single‐radiation group and the single‐drug group at 24 and 48 hours in H1299/H460 cells (Figure 3E‐F). In H1650 cells, the combination group showed increased apoptosis 48 hours after irradiation (Figure S1).

### 3.4 Radiosensitization effect of birinapant

**FIGURE 4:**
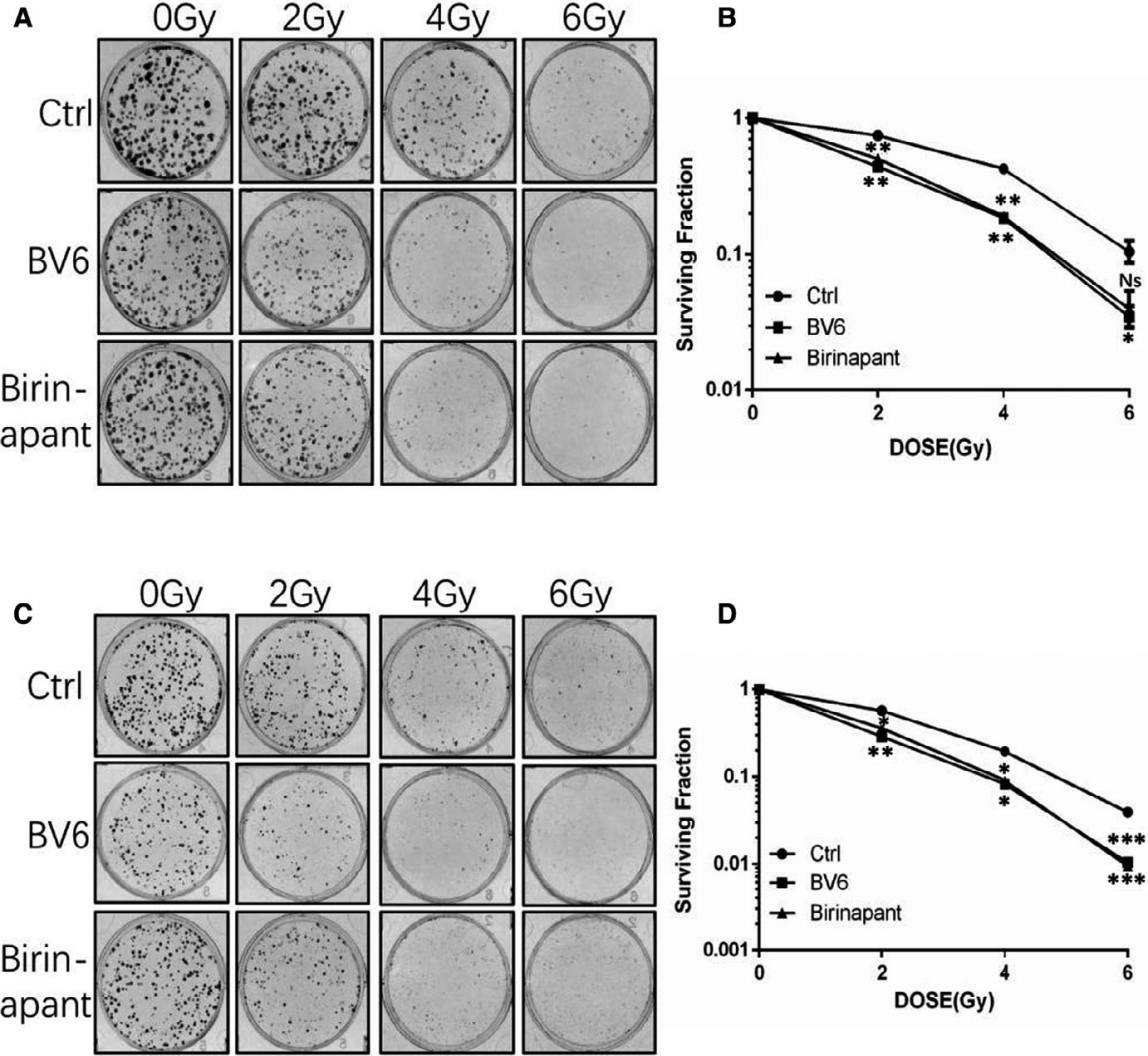
A, Representative images from the clone formation experiment of H1299 cells. B, The radiosensitizing effect of birinapant on H1299 cells was confirmed by clone‐forming cell‐survival test, by simulating the survival curve and calculating H1299 control group D_0_ = 2.04, experimental group D_0_ = 1.93 and 1.73, SER = 1.06 and 1.18. The asterisks above and below denote the Birinapant group and the BV6 group, respectively. C, Representative images from the clonogenic assay of H460 cells. D, The radiosensitizing effect of birinapant on H460 cells was confirmed by clone‐forming cell‐survival test, by simulating the survival curve and calculating H460 control group D_0_ = 1.54, experimental group D_0_ = 1.49 and 1.33, SER = 1.03 and 1.16. The asterisks above and below denote the Birinapant group and the BV6 group, respectively. All data shown are representative and have been repeated at least three times independently

The clone formation experiment was carried out to further verify the radiosensitization effect of birinapant. The results indicated that the sensitization of BV6 was better than birinapant at 2 Gy, while the two drugs had similar sensitization at 4 Gy (Figure 4). Consistent with previous reports, the two drugs have a slightly better sensitizing effect on H1299 cells than on H460, which may be attributed to H460 cells being more sensitive to radiation. In short, the significant sensitizing effect of birinapant on NSCLC cell line was proved through the calculation and comparison of SER (Refer to the Materials and methods for the rationale of SER).

### 3.5 Birinapant plays a radiosensitizing effect in a way that downregulates the level of CIAP1/2 protein

**FIGURE 5:**
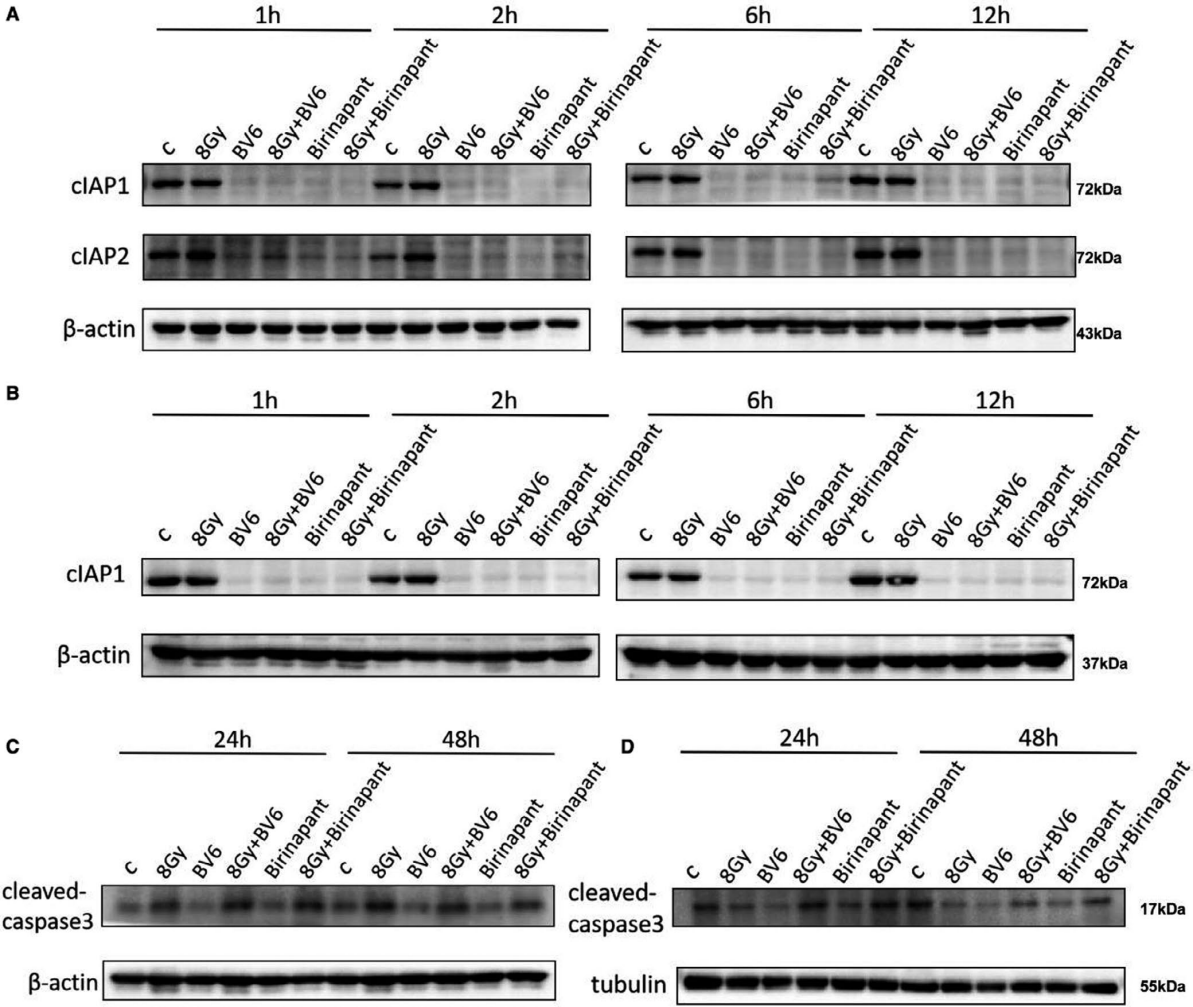
Protein expression for cells irradiated and exposed to birinapant, measuring (A) Protein expression of cIAP1/cIAP2 in H1299 cells; B, Protein expression of cIAP1 and caspase‐3 in H460 cells; C, Protein expression of cleaved‐caspase‐3 in H1299 cells; D, Protein expression of cleaved‐caspase‐3 in H460 cells. All data shown are representative and have been repeated at least three times independently

The effect of birinapant on cIAP1 and cIAP2 protein levels after exposure to IR was tested with a Western blot assay (Figure 5A‐B). In H1299 and H460 cells, cIAP1 and cIAP2 showed rapid degradation after about 60 minutes in cells treated with birinapant or BV6, whereas IR treatment alone did not have a significant effect. The degradation of cIAP1 by birinapant seems to be more significant than that of cIAP2. We also detected cleaved‐caspase3 at 24 and 48 hours (Figure 5C‐D), which was activated after 24 hours of exposure to birinapant or BV6 and after degradation of cIAP1 and cIAP2. As expected, birinapant activated more caspase3 by inhibiting the cIAP1/2 protein, thereby promoting more radiation‐induced apoptosis of tumour cells.

### 3.6 Birinapant upregulates cIAP1/2 gene expression

**FIGURE 6:**
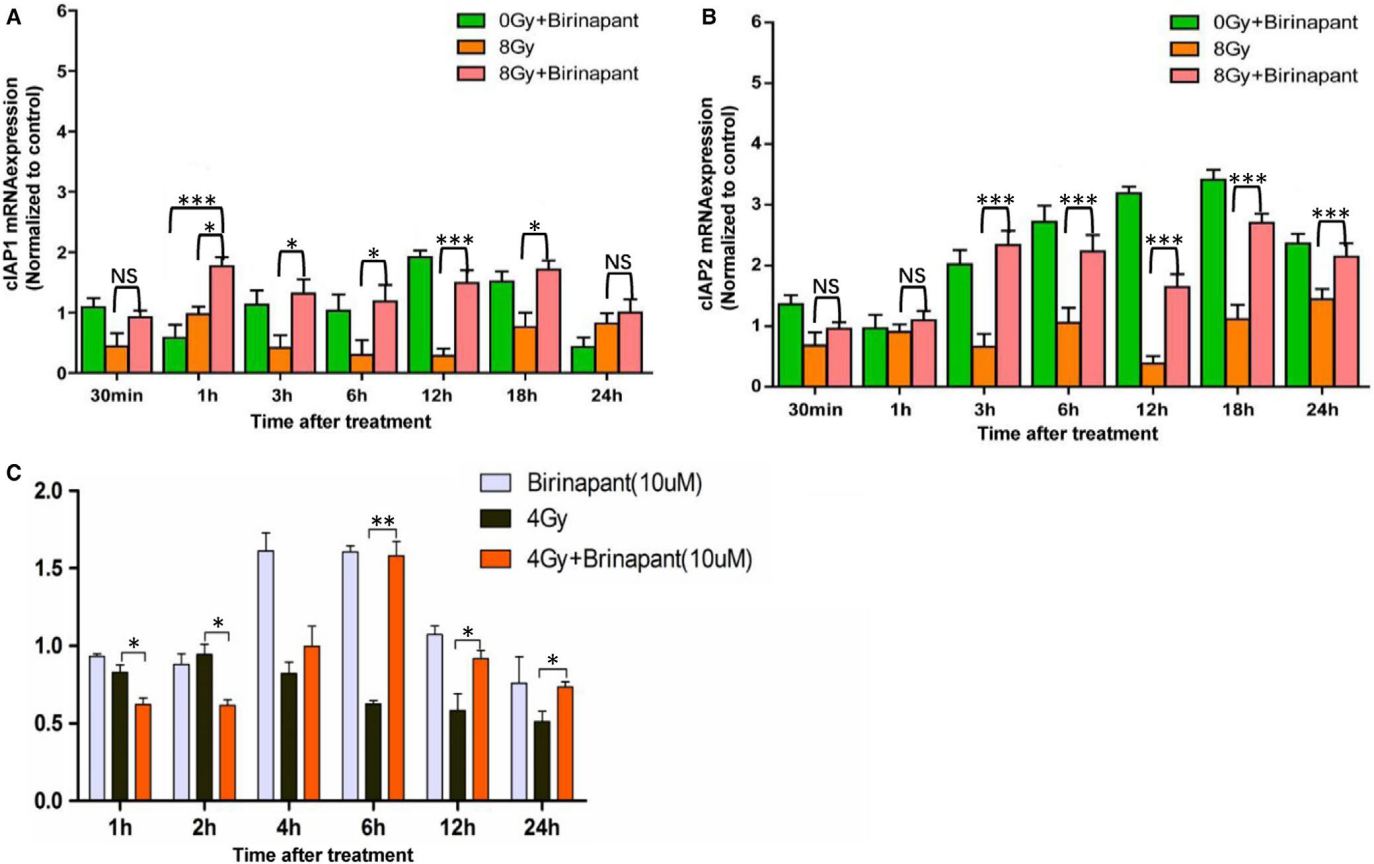
cIAP1/2 mRNA levels via RT‐PCR analysis at the indicated post‐irradiation times. A, mRNA levels of cIAP1 protein in H1299 cells; B, mRNA levels of cIAP2 protein in H1299 cells; C, mRNA level of cIAP1 protein in H460 cells. Error bars are means ± SD, n = 3 independent replicates and *P* < .05, *P* < .01, or *P* < .001 were considered statistically significant

cIAP1/2 mRNA levels were analysed by RT‐PCR (Figure 6). To our surprise, in H1299 cells, mRNA levels of cIAP1, and especially cIAP2, were elevated after treatment with birinapant (Figure 6A‐B). In contrast, cIAP1 mRNA levels within H460 cells showed a significant increase in the combination group compared with the single‐radiation group (Figure 6C). The mRNA expression levels of cIAP1/2 antagonistic related genes are shown in Figure S2. Gene expression levels of antagonistic genes, especially the Smac gene, decreased significantly after irradiation, which is consistent with the previously observed increase of cIAP1/2 gene levels. In summary, our results suggested that birinapant can downregulate cIAP1/2 protein levels, while upregulating their mRNA levels.

## 4 DISCUSSION

Radiotherapy is one of the main treatments for malignant tumours including lung cancer.^32^ However, the radiation tolerance of tumour cells has hindered the improvement of the efficacy of radiotherapy.^33^ For many years, humans have been trying to use radiosensitizers to reduce the radiation resistance of tumours and improve the efficacy of radiotherapy. The exploration of the mechanism of radiation tolerance of tumour cells plays a positive role in the development of radiosensitizers.

As previously mentioned, cIAP1/2, like XIAP, are members of the IAP protein family with an anti‐apoptotic effect.^17^ Since radiotherapy inhibits tumour growth by inducing apoptosis,^31^ we question whether IAP proteins also play a partial role in radiation‐resistant cells. The radiosensitivity of six NSCLC cell lines was first explored and then the expression of cIAP1/2 protein in these six cell lines was examined. Consistent with the hypothesis, there was a positive correlation between cIAP1/2 protein expression and radiation resistance. What is even more interesting is that radiation seems to induce the expression of cIAP protein to some extent. This indicates that cIAP1/2 plays a significant role in radiation resistance.

Second mitochondria‐derived activator of caspase is a natural antagonist of IAPs that exists in mitochondria. When cells are stimulated by apoptosis, mitochondria release Smac protein into the cytoplasm to bind to IAPs, making it lose the inhibition of caspase activity and subsequently promote cell apoptosis.^11^, ^20^, ^34^ Three previous papers published in Cell^35^, ^36^, ^37^ have reported that Smac mimetics can induce auto‐ubiquitination and cIAP1/2 degradation through different mechanisms, leading to tumour necrosis‐factor‐alpha‐mediated cancer cell death.^38^ These studies have prompted the introduction of Smac mimetics in anti‐cancer therapies. There have been many previous reports that IAP inhibitors, such as BV6, can be used as radiation sensitizers.^28^, ^29^, ^30^, ^39^, ^40^, ^41^ As a bivalent Smac mimetic, we attempted to verify whether birinapant also has similar effect and explored the preliminary mechanisms. The attempt was in line with the current trend of most pro‐apoptotic drugs combined with other therapies to increase the efficacy.

Sensitization ratio (SER) refers to the ratio of dose with the same biological effect in the presence or absence of drugs, which is an important standard to measure the effectiveness of sensitizers.^42^ We chose two NSCLC cell lines, H1299 and H460, which have very different radiosensitivities for follow‐up experiments. In the cell‐cloning experiment, the survival curves of the two kinds of cells under different treatment conditions were fitted, and the sensitization effectiveness of the two drugs was determined by calculating the sensitization ratio. The results showed that birinapant was similar to the positive control, BV6, and showed a significant sensitizing effect for both kinds of cells. Further research showed that birinapant is involved in the degradation of IAP protein. Although the H460 cell line does not express cIAP2 protein, the behaviour of cIAP1 and cIAP2 is similar. Moreover, with the addition of birinapant, the downstream caspase‐3, an apoptotic executive protein, was more activated. These results suggest that birinapant could be effective against NSCLC by those therapies. This is also consistent with previous reports where IAP antagonists induced apoptosis by targeting cIAP1/2.^6^, ^43^, ^44^ More specifically, birinapant relieves the radiation resistance of tumour cells by inhibiting cIAP1/2, relieving cIAPs‐mediated caspase inhibition, and then combined with radiation leads to more tumour cell apoptosis. Unexpectedly, we found that the mRNA level of cIAP1/2, however, increased compared with the control group. Although the effect of the drug on elevated cIAP1/2 mRNA levels has not been reported before, we theorize that this is a manifestation of cellular stress. Cellular stress means that when prokaryotic or eukaryotic cells are subjected to a variety of obvious environmental changes or stimuli, they can produce a series of adaptive changes, which eventually lead to changes in gene expression.^45^, ^46^, ^47^ When birinapant enters the cells, the cells show negative stress, that is, the expression of the cIAP1/2 gene increases, but due to the strong inhibition of cIAP1/2 protein by birinanpant, the translation of cIAP1/2 gene is blocked. As a result, the level of cIAP1/2 protein was suppressed and the level of mRNA was upregulated at the same time. Of course, further research on the ways of cIAP1/2 mRNA translation will reveal this interesting phenomenon more accurately.

## 5 CONCLUSION AND PROSPECTS

The present paper analysed the correlation between radiosensitivity of NSCLC and cIAP1/2 protein levels in six different NSCLC cell lines and found that cells are insensitive to radiation show high expression of cIAP1/2 protein. This supports the view that high expression of cIAP1/2 protein is a key factor that explains radiation resistance in NSCLC, providing the basis of a strategy to overcome radiation resistance in NSCLC cell lines. More importantly, our works confirmed that birinapant increased the radiosensitivity of NSCLC and promoted apoptosis by inhibiting the expression of cIAP/2 protein which provided more possibilities for the selection of sensitizers for clinical radiotherapy of NSCLC in the future.

## CONFLICT OF INTEREST

The authors declare no conflict of interest.

## AUTHOR CONTRIBUTIONS

**Hao Sun:** Conceptualization (equal); Data curation (lead); Formal analysis (lead); Methodology (lead); Visualization (lead); Writing‐original draft (lead). **Yanan Du:** Conceptualization (equal); Data curation (equal); Formal analysis (lead); Methodology (lead); Software (equal); Validation (lead); Visualization (equal); Writing‐original draft (lead). **Ming Yao:** Conceptualization (equal); Data curation (equal); Formal analysis (equal); Methodology (equal); Validation (equal). **Qin Wang:** Methodology (supporting). **Kaihua Ji:** Funding acquisition (supporting); Methodology (supporting). **Liqing Du:** Methodology (supporting); Visualization (supporting). **Chang Xu:** Methodology (supporting); Visualization (supporting). **Ningning He:** Formal analysis (supporting); Funding acquisition (supporting). **Jinhan Wang:** Funding acquisition (supporting); Methodology (supporting); Software (supporting). **Manman Zhang:** Funding acquisition (supporting); Methodology (supporting). **Yang Liu:** Formal analysis (supporting); Methodology (supporting). **Yan Wang:** Conceptualization (lead); Funding acquisition (lead); Methodology (equal); Project administration (equal); Validation (equal); Writing‐review & editing (lead). **Kaixue Wen:** Funding acquisition (equal); Methodology (equal); Supervision (equal); Writing‐review & editing (equal). **Qiang Liu:** Conceptualization (lead); Funding acquisition (lead); Methodology (equal); Project administration (equal); Validation (equal); Visualization (equal); Writing‐review & editing (lead).

## Supporting information

Fig S1‐S2

## Data Availability

The data that support the findings of this study are available from the corresponding author upon reasonable request.
