# Supplementary material for: cIAP1/2 are involved in the radiosensitizing effect of birinapant on NSCLC cell line in vitro
Source: J Cell Mol Med. 2021 May 3;25(13):6125–36. doi: 10.1111/jcmm.16526 (PMC8366455; doi:10.1111/jcmm.16526)
Supplement: Supplementary file 1 — Fig S1‐S2 [file JCMM-25-6125-s001.docx]

**Supplementary Materials:**

**

**

**Figure** s1 Effect of birinapant Combined Radiation on Apoptosis of H1650 Cells. Error bars are means ± SD, n = 3 independent replicates and *P* <0.05, *P* <0.01, or *P* <0.001 were considered statistically significant.


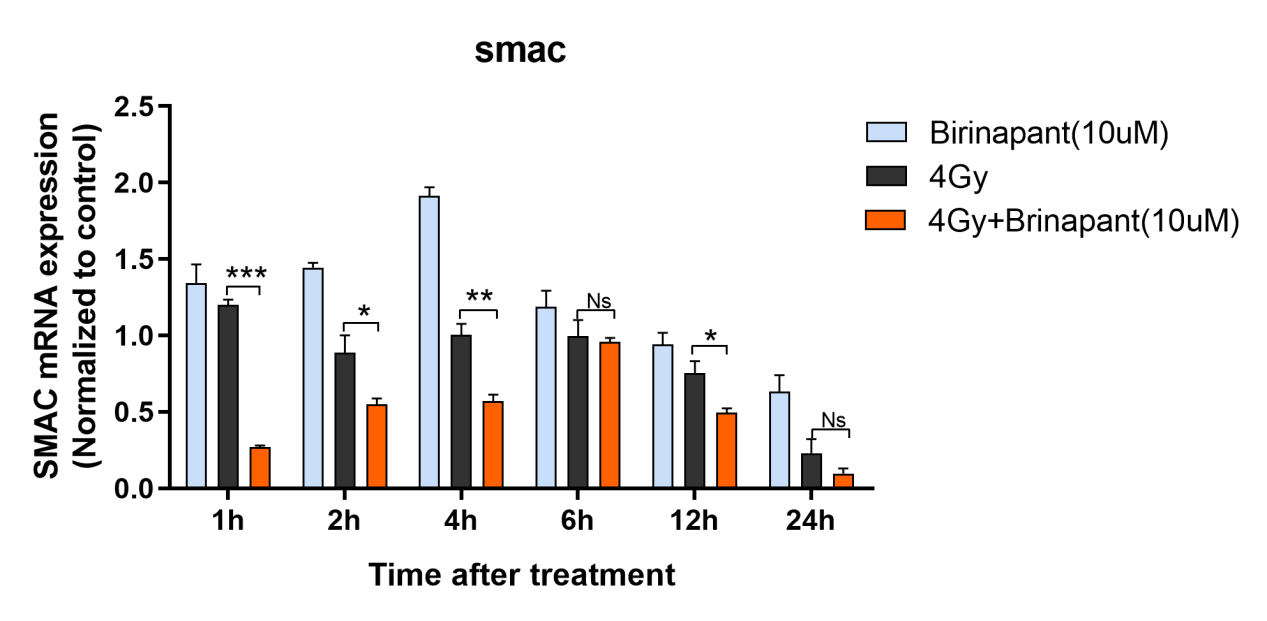

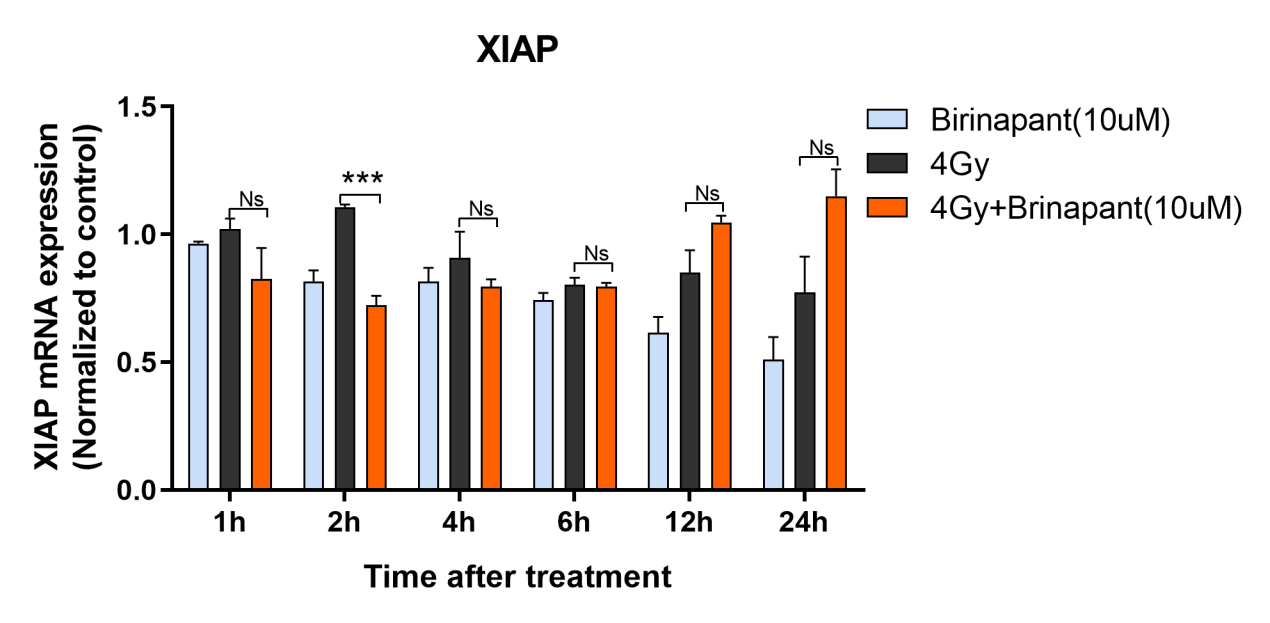


**Figure** s2 Effect of birinapant Combined Radiation on the mRNA Expression of Apoptosis-related Gene in H460. Error bars are means ± SD, n = 3 independent replicates and *P* <0.05, *P* <0.01, or *P* <0.001 were considered statistically significant.
